# Supplementary figures and images for: [18F]MK-7246 for Positron Emission Tomography Imaging of the Beta-Cell Surface Marker GPR44
Source: Pharmaceutics. 2023 Feb 2;15(2):499. doi: 10.3390/pharmaceutics15020499 (PMC9962486; doi:10.3390/pharmaceutics15020499)

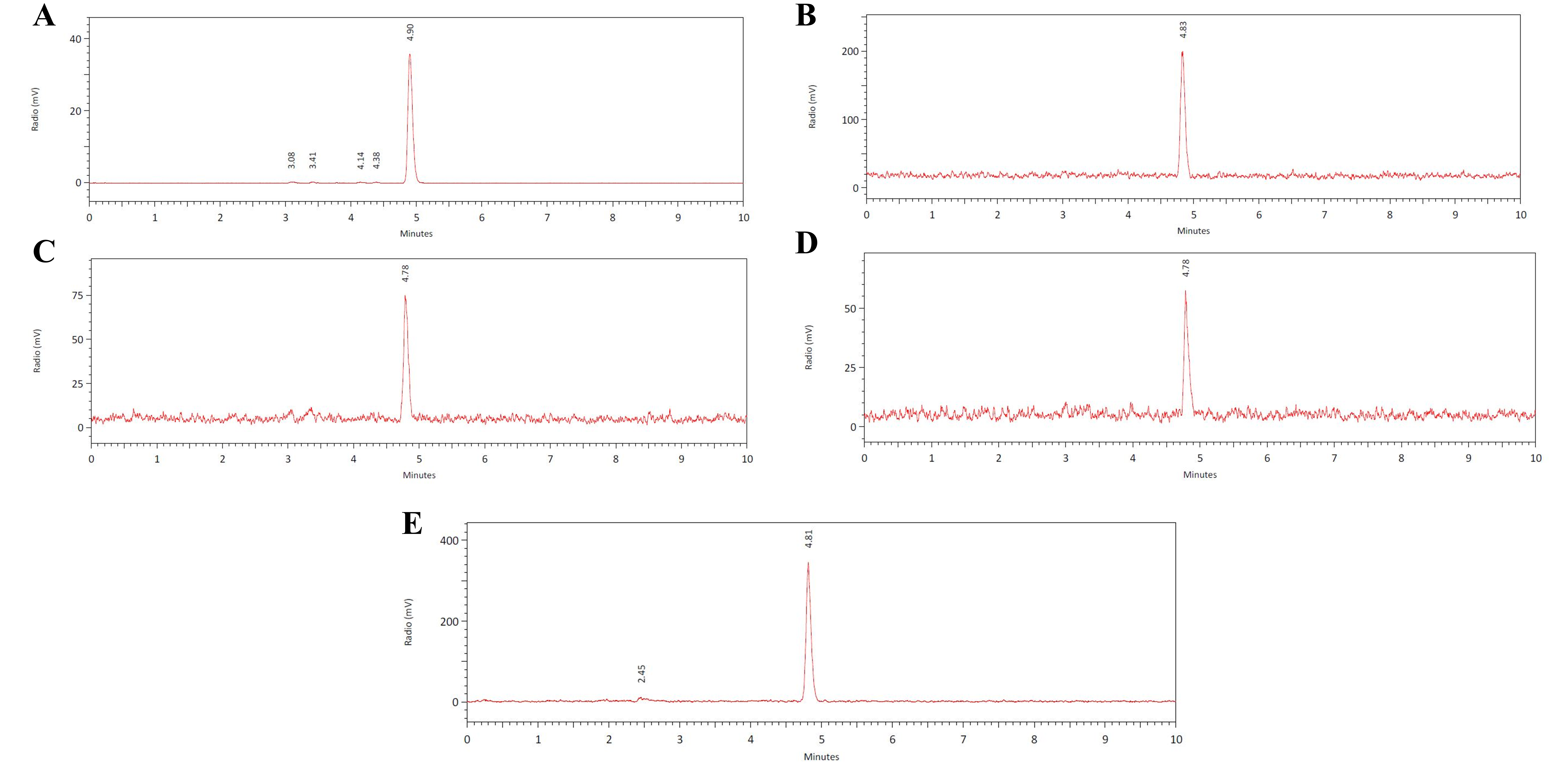

Supplement: Supplementary file 1 [file pharmaceutics-15-00499-s001.zip › pharmaceutics-2112647-supplementary.tif]
